# Identification of DNA Damage Repair-Associated Prognostic Biomarkers for Prostate Cancer Using Transcriptomic Data Analysis

**DOI:** 10.3390/ijms222111771

**Published:** 2021-10-29

**Authors:** Pai-Chi Teng, Shu-Pin Huang, Chia-Hsin Liu, Ting-Yi Lin, Yi-Chun Cho, Yo-Liang Lai, Shu-Chi Wang, Hsin-Chih Yeh, Chih-Pin Chuu, Deng-Neng Chen, Wei-Chung Cheng, Chia-Yang Li

**Affiliations:** 1 Taipei City Hospital Renai Branch, Taipei 10629, Taiwan; paichi.teng@gmail.com; 2 Department of Urology, School of Medicine, College of Medicine, Kaohsiung Medical University, Kaohsiung 80708, Taiwan; shpihu73@gmail.com (S.-P.H.); patrick1201.tw@yahoo.com.tw (H.-C.Y.); 3 Department of Urology, Kaohsiung Medical University Hospital, Kaohsiung Medical University, Kaohsiung 80708, Taiwan; 4 Graduate Institute of Clinical Medicine, College of Medicine, Kaohsiung Medical University, Kaohsiung 80708, Taiwan; 5 Ph.D. Program in Environmental and Occupational Medicine, College of Medicine, Kaohsiung Medical University, Kaohsiung 80708, Taiwan; 6 Research Center for Cancer Biology, China Medical University, Taichung 40403, Taiwan; b881642@gmail.com (C.-H.L.); demicho.0111@gmail.com (Y.-C.C.); 7 Department of Medical Research, Taipei Veterans General Hospital, Taipei 11217, Taiwan; lintingyi2014@gmail.com; 8 Graduate Institute of Biomedical Science, China Medical University, Taichung 40403, Taiwan; yolianglai@gmail.com; 9 Department of Radiation Oncology, China Medical University Hospital, Taichung 40403, Taiwan; 10 Department of Medical Laboratory Science and Biotechnology, Kaohsiung Medical University, Kaohsiung 80708, Taiwan; shuchiwang@kmu.edu.tw; 11 Department of Urology, Kaohsiung Municipal Ta-Tung Hospital, Kaohsiung 80145, Taiwan; 12 Institute of Cellular and System Medicine, National Health Research Institutes, Miaoli 35053, Taiwan; cpchuu@nhri.edu.tw; 13 Department Management Information Systems, National Pingtung University of Science and Technology, Pingtung 91201, Taiwan; dnchen@mail.npust.edu.tw; 14 Ph.D. Program for Cancer Biology and Drug Discovery, China Medical University and Academia Sinica, Taichung 40403, Taiwan; 15 Graduate Institute of Medicine, College of Medicine, Kaohsiung Medical University, Kaohsiung 80708, Taiwan

**Keywords:** prostate cancer (PC), DNA damage repair (DDR), DDR-based transcriptomic biomarker, prognostic marker, cancer survival

## Abstract

In the recent decade, the importance of DNA damage repair (DDR) and its clinical application have been firmly recognized in prostate cancer (PC). For example, olaparib was just approved in May 2020 to treat metastatic castration-resistant PC with homologous recombination repair-mutated genes; however, not all patients can benefit from olaparib, and the treatment response depends on patient-specific mutations. This highlights the need to understand the detailed DDR biology further and develop DDR-based biomarkers. In this study, we establish a four-gene panel of which the expression is significantly associated with overall survival (OS) and progression-free survival (PFS) in PC patients from the TCGA-PRAD database. This panel includes *DNTT*, *EXO1*, *NEIL3*, and *EME2* genes. Patients with higher expression of the four identified genes have significantly worse OS and PFS. This significance also exists in a multivariate Cox regression model adjusting for age, PSA, TNM stages, and Gleason scores. Moreover, the expression of the four-gene panel is highly correlated with aggressiveness based on well-known PAM50 and PCS subtyping classifiers. Using publicly available databases, we successfully validate the four-gene panel as having the potential to serve as a prognostic and predictive biomarker for PC specifically based on DDR biology.

## 1. Introduction

Prostate cancer (PC) is the second most commonly diagnosed cancer in men with an estimate of 1.4 million new cases, causing approximate 374,000 deaths worldwide in 2020 [1]. Androgen deprivation therapy has been the mainstay of systemic treatment for PC; however, many patients eventually develop metastasis and resistance to androgen deprivation therapy, and the transition to metastatic castration-resistant PC (mCRPC) represents a lethal evolution of PC. In the recent decade, the advent of next-generation hormonal therapies such as abiraterone [2], enzalutamide [3], apalutamide [4], and darolutamide [5] has significantly impacted the treatment paradigm and survival of PC patients. Other treatment options for advanced PC include taxanes, antibody–drug conjugates, radium-223, DNA damage repair (DDR) inhibitors, etc [6]. Of note, olaparib, a poly (adenosine diphosphate [ADP]-ribose) polymerase (PARP) inhibitor, was approved in 2020 as the first targeted therapy against a specific molecular phenotype of mCRPC with certain DDR gene mutations [7,8]. 

Many studies have reported both somatic and germline mutations of DDR pathways in PC. For example, The Cancer Genome Atlas (TCGA) dataset revealed the inactivation of DDR genes including *BRCA1*, *BRCA2*, *CDK12*, *ATM*, *FANCD2*, and *RAD51C* in 19% of tissues collected from 333 localized PC patients who underwent radical prostatectomy [9]. A systemic review by Lang et al. summarized 11,648 records from 80 studies and demonstrated that the median prevalence rates for somatic and germline mutations of DDR genes were 10.7% and 18.6%, respectively [10]. Among these, *BRCA2*, *ATM*, and *PALB2* genes had higher mutation rates of ≥4% [10], while in mCRPC, 19.3% of aberrations in *BRCA2*, *BRCA1*, and *ATM* genes were found in bone or soft tissue tumor biopsies that were substantially more frequently compared to those in primary PC tissues [11]. 

Certain DDR genes such as *BRCA1*, *BRCA2*, and *ATM* encode proteins that are essential for repairing DNA double-strand breaks by homologous recombination repair (HRR) [12,13]. The mutations involved in these genes can lead to carcinogenesis. DNA single-strand breaks are repaired by PARP enzymes (i.e., “nicks” in the DNA). PARP inhibitors can trap PARP enzymes at sites of DNA nicks and inactivate the repair of single-strand breaks that ultimately progress to double-strand breaks [14]. It was observed that *PARP1*^−/−^ mice did not develop early-onset malignancy, but *BRCA2*-deficient cells were sensitive to PARP inhibitors [15], so the rationale and application of adding PARP inhibitors such as olaparib to cause death of tumor cells especially with defective HRR capacity has been developed in treatment for cancers including PC [16]. 

The results of the PROfound trial [7,8], which categorized the enrolled patients into cohort A who had at least one mutation in *BRCA1*, *BRCA2*, or *ATM* genes and cohort B who had at least one mutation in any of the other 12 genes, led to the FDA approval of olaparib use in mCRPC with one of the 15 mutated HRR genes including *BRCA1*, *BRCA2*, *ATM*, etc. Interestingly, a clinical benefit with statistical significance was not observed in the patients of cohort B receiving olaparib, while the patients of cohort A had significantly longer median progression-free survival (PFS) and overall survival (OS) with olaparib administration [7]. This observation highlights the need to delineate the genomic indicators and further understand the detailed biology of DDR specifically for PC.

In addition to HRR, there are at least five other major DDR pathways such as base excision repair, nucleotide excision repair, mismatch repair, non-homologous end-joining, and Fanconi anemia DNA repair pathways [17,18,19]. In this study, we aimed to identify genes that are significantly associated with these DDR pathways and examine their contributions to survival in PC using our previously developed user-friendly platform, i.e., DriverDBv3 [20]. It was found that the expression of a four-gene panel including *DNTT*, *EXO1*, *NEIL3*, and *EME2* was the most significantly associated with OS of PC patients through a rigorous bioinformatic process; moreover, gene expressions were examined based on two well-established PC classifiers, i.e., PAM50 [21] and PCS [22,23], and it was found that the expression of the four-gene panel was significantly correlated with aggressiveness based on PAM50 and PCS subtypes, both of which have been validated for their clinical significance including prognostication and prediction of treatment response in PC [24,25]. These findings demonstrate the potential application of a DDR pathway-based transcriptomic panel as a prognostic and predictive biomarker in PC.

## 2. Results

### 2.1. Identification of DDR Genes Associated with Overall Survival in Prostate Cancer

**Figure 1:**
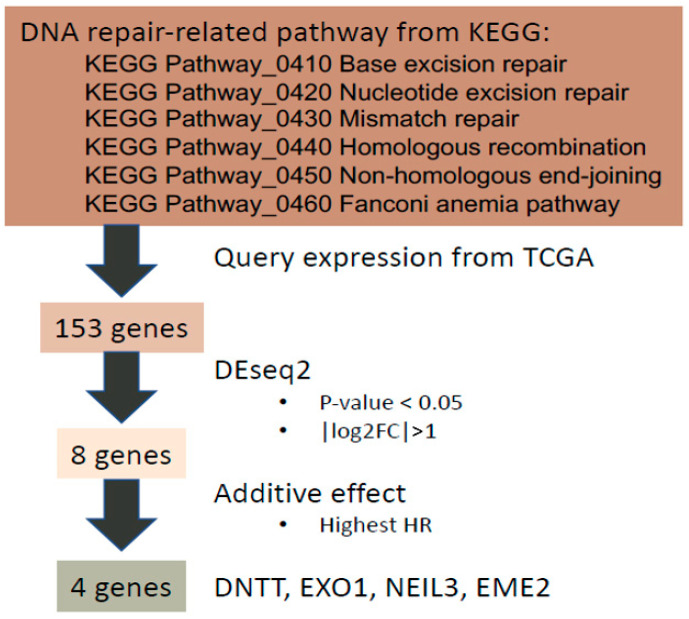
The workflow of developing our 4-gene panel as a transcriptomic biomarker for PC prognosis.

Firstly, genes related to six major DDR pathways in the Kyoto Encyclopedia of Genes and Genomes (KEGG) pathway database [26,27] were examined including base excision repair, nucleotide excision repair, mismatch repair, HRR, non-homologous end-joining repair, and Fanconi anemia DNA repair pathways (Figure 1). A total of 154 genes involved in DDR pathways in the KEGG database were identified and transcriptomic profiling of 153 genes with associated clinical data exported from TCGA-PRAD [9] through our previously developed DriverDBv3 platform [20] (Supplementary Table S1). One of the 154 genes, *GTF2H2C_2*, did not have expression data and was therefore excluded. The individual expression of the 153 DDR-associated genes was examined in PC and benign prostate tissue. Significantly differentially expressed (SDE) genes were selected if their absolute value of log_2_ fold change was greater than 1 (i.e., | log_2_ FC | >1) with *p* value of less than 0.05. Consequently, eight SDE genes were identified, including *RAD51*, *RPA4*, *SLX1B*, *POLN*, *EME2*, *EXO1*, *DNTT*, and *NEIL3*. The corresponding survival data of the eight SDE genes including hazard ratio (HR) of OS and p value are shown in Table 1.

**Figure 2:**
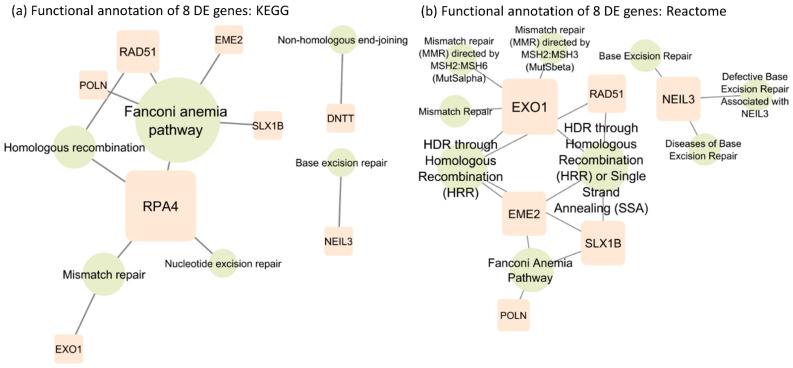
(**a**) Functional annotation of 8 SDE genes based on the KEGG database. (**b**) Functional annotation of 8 SDE genes based on the Reactome database.

The functional annotation of the eight SDE genes was investigated using the KEGG pathway [27] (Figure 2a) and Reactome pathway databases [28] (Figure 2b). The KEGG analysis showed three common repair pathways enriched by a six-gene network, from genes required for inducing double-strand break formation (*EME2*), DNA intercross link-strand unhooking (*SLX1B*) to excision of mispaired DNA (*EXO1*), guiding homologous recombination (*RAD51*, *RPA4*) to DNA resynthesis (*POLN*). The pathway enrichment showed that five genes were present in the Fanconi anemia pathway, a generalized pathway encompassing sequential steps critical in DNA repair. 

Enriched pathways in mismatch repair, homologous recombination and Fanconi anemia serve as critical genomic stability/integrity regulators. The other analysis in Reactome further identified the enriched genes as being involved in deregulations of initial steps in mismatch repair by the *MSH2/6* and *2/3* complex. Consistent with the KEGG analysis, the Reactome analysis also identified a second DNA repair pathway from mismatch repair. *NEIL3* enriched pathways involved in base excision repair differ from mismatch repair in purpose, method, and enzymes involved. Contrary to mismatch repair that corrects misincorporation during synthesis, base excision repair restores oxidative bases from oxidative reactions and basic sites that result from ionizing radiation, heat, and spontaneous base loss. However, the *DNTT* gene is not annotated in the Reactome database. All the other signaling pathways involving the eight SDE genes are illustrated in Supplementary Figure S1.

### 2.2. Identification of a Four-Gene Panel as Prognostic Markers for PC Survival

**Figure 3:**
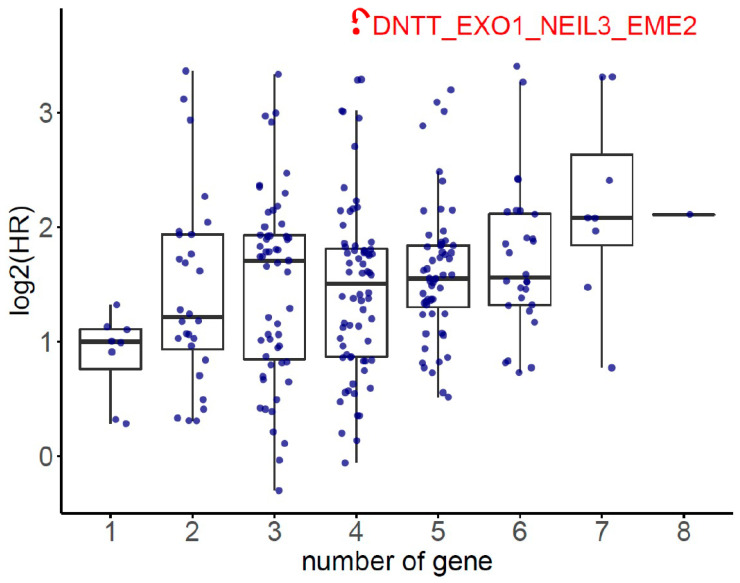
The additive effect on hazard ratio (HR) of overall survival (OS) for each combination of any of the 8 SDE genes.

**Figure 4:**
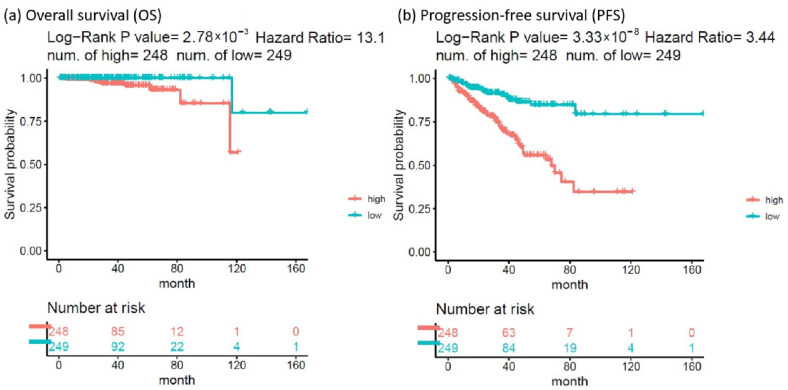
Kaplan–Meier analysis of (**a**) overall survival and (**b**) progression-free survival (PFS) in patients with higher vs. lower expression of the 4-gene panel (*DNTT*, *EXO1*, *NEIL3*, and *EME2*).

The HR of the eight SDE genes in PC survival analysis was examined using TCGA-PRAD database. There were 497 patients who had OS and transcriptomic data of the eight SDE genes listed in Table 1. To obtain an optimal panel of genes that had the greatest contribution to prognostication, the additive effect of any combination of the eight SDE genes on HR of OS was investigated. As the expression of *DNTT* gene is downregulated in PC compared to benign prostate tissue (Table 1), the log_2_ expression of *DNTT* gene was multiplied by minus one for the analysis of additive effect. As the median Z score of each gene combination was set as the cutoff, the patients were grouped into higher or lower expression groups. As shown in Figure 3, higher Z scores of *DNTT*, *EXO1*, *NEIL3*, and *EME2* genes resulted in the highest HR of OS among all the 255 combinations, while Kaplan–Meier analysis demonstrated that the patients with higher expression of the four-gene panel (i.e., *DNTT*, *EXO1*, *NEIL3*, and *EME2*) were significantly associated with poorer OS compared to the patients with lower expression (log-rank HR = 13.1, 95% confidence interval (CI) = 1.59 to 108.03, *p* = 0.0028, Figure 4a). The patients with higher expression of the four-gene panel also had significantly worse PFS compared to those with lower expression (log-rank HR = 3.44, 95% CI = 2.16 to 5.49, *p* < 0.0001, Figure 4b). The Kaplan–Meier plot of individual genes is illustrated in Supplementary Figure S2.

### 2.3. Multivariate Cox Regression Analysis

A multivariate Cox regression model [29] was conducted to adjust possible clinical confounding variables including age (years), baseline prostate-specific antigen (PSA) levels (ng/mL), TNM stages (T1N0M0 or T2N0M0 vs. others), and Gleason scores (≤7 vs. >7). Notably, there were only three M1 (i.e., PC with distant metastasis) patients in this study because all the PC tissues were obtained from primary PC by radical prostatectomy. As shown in Table 2, the patients with higher four-gene expression were independently associated with poorer OS (Cox HR = 13.8, 95% CI = 1.21 to 158, *p* = 0.0348) and PFS (Cox HR = 2.32, 95% CI = 1.36 to 3.95, *p* = 0.0020). The patients with Gleason scores >7 had significantly worse PFS compared to those with Gleason scores ≤7 (Cox HR = 2.71, 95% CI = 1.61 to 4.56, *p* = 0.0002). Otherwise, the remaining clinical variables did not reach statistical significance in this multivariate Cox regression model.

### 2.4. Expression of the Four-Gene Panel Based on Luminal and Basal Subtypes of PC

**Figure 5:**
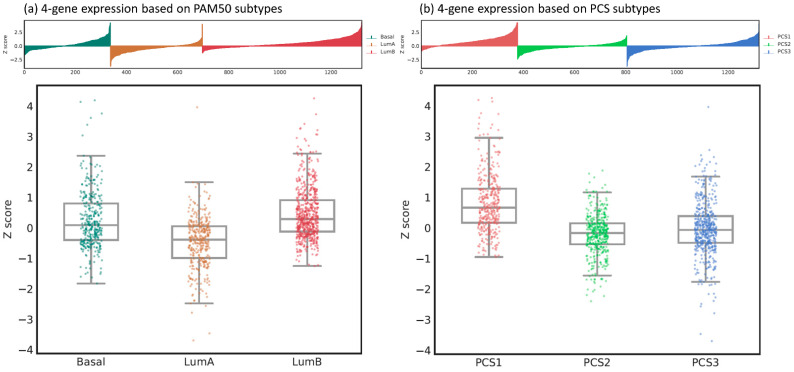
The expression of the 4-gene panel based on (**a**) PAM50 and (**b**) PCS subtypes.

PAM50 is a 50-gene panel that can categorize PC into luminal A, luminal B, and basal subtypes [21]. Among them, luminal B is the most aggressive and luminal A is the least aggressive subtype. The clinical significance of PAM50 classifier has been extensively validated in many studies and clinical trials [21,24,30,31,32,33,34]. Prostate Cancer Classification System (PCS) is another luminal–basal subtyping method that classifies PC into PCS1, PCS2 and PCS3 based on a 37-gene panel [23]. PCS1 is associated with the worst survival, the most resistance to antiandrogen therapy and independence of AR canonical pathway, whereas PCS2 is the least aggressive subtype [23,25]. In this study, we examined the expression of our four-gene panel based on these two well-known PC classifiers using data from an independent cohort (i.e., the DISC cohort) [23]. The analysis of gene expression based on PAM50 and PCS subtypes was conducted using the public Prostate Cancer Transcriptome Atlas (PCTA, http://www.thepcta.org/; accessed date 15 September 2021) platform. The Z scores of our four-gene panel were significantly associated with PAM50 (one-way ANOVA test F value = 133.986, *p* < 0.001, Figure 5a) and PCS (one-way ANOVA test F value = 168.826, *p* < 0.001, Figure 5b) subtypes. In terms of the PAM50 system, luminal B and luminal A had the highest and lowest Z scores, respectively. In the PCS system, PCS1 had the highest Z scores, whereas PCS2 had the lowest Z scores. The expression of our four-gene panel was consistent with the aggressiveness with respect to either PAM50 or PCS classifiers.

## 3. Discussion

Aberrations of the AR signaling pathway are a hallmark of prostate carcinogenesis and thus therapies targeting AR have been the basis of PC treatments for many decades. However, it has been revealed that many other genetic changes either lead to or result in dysregulation of AR transcriptomic activity [14]. When cells with these molecular changes have defective ability of DNA repair, these permanent genomic insults will further worsen PC patients’ survival and treatment response. Some researchers have also reported a positive feedback circuit between AR activity and DDR [35]. Rearrangements involving *TMPRSS2*, which is regulated by androgen, are shown to contribute to prostate carcinogenesis as well [36], and these structural rearrangements such as *TMPRSS2-ERG* translocation might result in AR-related DNA double-strand breaks [37,38,39,40]. Miller et al. found that chromosomal instability is highly indicative of metastatic potential, antiandrogen resistance and PC all-cause mortality, and is associated with the signaling pathways involving regulation of centrosomes, chromosomal segregation and assembly of mitotic spindles [41]. The functional annotation in our study also implied interactions between DDR and other biological pathways like cell cycle check points, TP53 activity, and DNA glycosylase (Supplementary Figure S1). As DDR biology is more understood, a well-developed, clinically meaningful and DDR-based biomarker could shed light into the realization for precision medicine of using DDR-targeted therapies in PC.

There have been various clinical parameters, biomarkers or commercial platforms developed for prognostication and prediction of treatment response in PC. Examples include cancer of the prostate risk assessment (CAPRA) scores [42], Decipher [43], Prolaris [44], PAM50 [21], and PCS [23]. Liquid biopsies including circulating tumor DNA (ctDNA), circulating tumor cells (CTCs) and extracellular vesicles (EVs) have also been extensively developed as a non-invasive method because they do not require tissue sampling. Of note, utilization of CTCs has been applied in PC clinical trials and even included in The Prostate Cancer Working Group 3 (PCWG3) consensus [45,46,47]. Moreover, the transcriptomic and protein expression of AR splice variant 7 (*AR-V7*) in CTCs was reported to be useful in selecting PC patients who could benefit from next-generation hormonal therapies [48]. EVs collected from PC patients’ urine or plasma also hold the potential to be a promising biomarker [46,49]. However, very few PC biomarkers are specifically derived from DDR biology, since the first DDR-based therapy, i.e., olaparib, was just approved in May 2020 for PC; consequently, there is a clinically unmet need of DDR-based biomarkers in PC. This study provides novel insights into the transcriptomic biomarker that could indicate PC prognostication reflective of DDR biology, resulting in a four-gene panel that consists of *EXO1, DNTT, NEIL3* and *EME2*. 

*EXO1* interacts with *MSH2* and participates in DNA mismatch repair, HRR, cell cycle checkpoints, and replication fork maintenance [50,51]. Luo et al. demonstrated that *EXO1* expression is associated with the PFS and OS in PC [52]. Hua et al. identified nine RNA binding protein genes including *EXO1* that have prognostic vales in PC [53]. The genetic polymorphism of *EXO1* was found to elevate PC risks as well [54]. *EXO1* also plays an essential role in hereditary nonpolyposis colorectal cancer and its atypical forms [55]. Recently, *EXO1* was reported as being involved in non-homologous end joining and contribute to drug resistance in ovarian cancer [56]. Although the role of *DNTT* (also known as *TdT*) in PC is unclear, it has been recognized as an important marker in hematologic malignancies [57,58]. Merkel cell carcinoma, a rare but aggressive skin cancer, was also shown to have higher positive rate of TdT protein expression [59]. *NEIL3* can secure mitotic chromosome segregation by repairing telomere damage [60], although deficiency of *NEIL3* was reported to enhance resistance to chemotherapy in PC [61]. Alterations of *NEIL3* are also associated with somatic mutation loads, carcinogenesis, and poor survival in many human cancers [62,63,64]. *EME2* can promote restart of replication fork and the genetic change of *EME2* and is therefore associated with DDR in malignancies [65,66]. These biological implications of our four-gene panel account for its success of prognostication in PC. It is noteworthy that *EXO1* and *EME2* are correlated with homologous recombination repair pathway in the functional annotation based on the Reactome database (Figure 2b). Several studies have reported that olaparib can inhibit the activity of *EXO1* protein leading to inactivation of PARP enzymes [67,68,69], whereas there is currently no study that reports the direct interaction between PARP inhibitors and *EME2*.

Although the Kaplan–Meier analyses revealed statistical significance of our four-gene panel in terms of both OS and PFS, there may be clinical confounding variables that led to bias in the analyses. To overcome this issue, a multivariate Cox regression model adjusting for commonly seen clinical variables in PC was conducted and the experimental results indicated that age and baseline PSA levels were not significant variables in this model. Baseline PSA levels have been considered inappropriate as a prognostic or predictive biomarker alone [70] and must be used along with other clinical parameters for risk stratification [71]. TNM stage has been utilized in many malignancies but heterogeneity has been widely reported even in patients with same staging [72]. In this study, there were only three M1 PC patients (i.e., with distant metastasis), whereas most of the patients were M0 (i.e., without distant metastasis); therefore, it is rational to conclude that patients of staging other than T1N0 or T2N0 possess a trend of having greater HR of OS and PFS despite not reaching statistical significance. With respect to Gleason scores, it is reasonable that Gleason scores were correlated with the PFS but, as treatment options for advanced PC have grown expeditiously [6], the survival of patients with advanced PC has become much prolonged and the initial Gleason scores of primary tumors might not be sufficient to predict OS for PC patients. In particular, when PC patients develop distant metastasis, many other clinical characteristics are more accurate and important for prognostication such as castration sensitivity [73], metastatic site [74], number of metastatic tumors [75], and prior lines of therapy [76]. This might explain why Gleason scores in our Cox regression model did not show significance in the OS analysis. Most importantly, the expression of our four-gene panel was independently associated with both OS and PFS with statistical significance.

As the concept of luminal or basal types has been extensively accepted to describe the aggressiveness of cancer in the field of pathology, a molecular signature that can represent luminal–basal biology was also established to better classify cancers. Initially, PAM50 was developed for categorizing breast cancer into normal, luminal A, luminal B, basal and HER2 subtypes [77,78]. Since PC is also a sex-hormone-dependent malignancy, Zhao et al. successfully applied PAM50 into PC but excluded normal and HER2 subtypes [21]. They also applied PAM50 to other carcinomas including adrenocortical, pancreatic, kidney, lung cancers, etc. with clinical significance [79]. PCS is another famous luminal–basal classifier that was directly derived from multiple PC datasets [23]. Yoon et al. have demonstrated the similarity and comparability between PAM50 and PCS classifiers [22]. The expressions of our four-gene panel highly correlated with aggressiveness of both PAM50 and PCS subtypes in an independent cohort (i.e., DISC cohort). This result makes the significance of our panel more convincing.

In conclusion, the need of new biomarkers that can reflect DDR biology will be rapidly increased for not only PC but also many other malignancies because of the advent of DDR-targeted treatments. We successfully demonstrated a rigorous bioinformatic pipeline using publicly available platforms and databases including TCGA-PRAD, KEGG, DriverDBv3, and PCTA to develop a DDR-based panel that could aid with PC prognostication. The clinical and biological associations of the four-gene expression were consistent and rational between different datasets. Our study suggested that this four-gene panel consisting of *EXO1, DNTT, NEIL3* and *EME2* is a promising DDR-derived biomarker that should be further explored to investigate its application and effectiveness in larger clinical studies.

## 4. Materials and Methods

### 4.1. Patient Clinical Information and Gene Expression Data Source

Gene expression (RNA-seq) profiles and sample clinical profiles were curated from DriverDBv3 database and samples of TCGA-PRAD were downloaded [20]. In total, 497 patients were included in this study. The baseline characteristics of patients are shown in Supplementary Table S2.

### 4.2. DNA-Repair Related Gene Selection

Six DNA-repair-relevant KEGG pathways were selected and a total of 154 genes belonging to these pathways were curated (Supplementary Table S1). *GTF2H2C_2* was not found in the TCGA-PRAD gene expression profile and was therefore discarded. In total, 153 gene expression profiles of curated genes were used for further analysis.

### 4.3. Differential Expression Analysis

Genes differentially expressed between the normal and tumor part were analyzed by DESeq2 algorithm [80]. Genes with an absolute value of log_2_-transform fold change greater than 1 and a *p*-value lower than 0.05 were considered statistically significant candidates, named SDE genes. Finally, 8 SDE genes were considered differentially expressed in PRAD.

### 4.4. Functional Annotation

Functional annotation of 8 SDE genes was conducted in the DriverDBv3 webserver [20]. Both the KEGG pathway database [27] and the Reactome pathway knowledgebase [28] were used to annotate genes with their associated functions, respectively.

### 4.5. Additive Effect Analysis

Additive effect of candidate genes was used to evaluate the synergic effect of combining multiple genes. Firstly, gene expression values were normalized by the z-score transformation for each gene, respectively. Secondly, the multi-gene score was calculated as the sum of normalized expression values of genes, and all possible combinations of 8 SDE genes were calculated. Thirdly, for each combination, patients were classified into two groups according to the median value of the multi-gene score, and survival analysis was conducted. Finally, the multi-gene combination with the lowest *p*-value was reported (Figure 3).

### 4.6. Survival Analysis

Univariate and multivariate Cox proportional hazards models were conducted by the “survival” library of R v4.0.1 package. Two clinical end points, OS and PFS, were analyzed. Four clinical and baseline characteristics (Supplementary Table S2) were considered as covariates in the multivariate Cox proportional hazards model: (1) patient’s age in year as a continuous variable; (2) prostate-specific antigen (PSA, ng/mL) value as a continuous variable; (3) Gleason score divided by two groups (≤7 vs. >7) as a categorial variable; and (4) TNM stage divided by two groups (T1/T2N0M0 vs. others) as a categorial variable. A *p*-value smaller than 0.05 was considered statistically significant.

## Figures and Tables

**Table 1:** The log_2_ fold change and corresponding hazard ratio (HR) of overall survival (OS) for each significantly differentially expressed (SDE) gene.

SDE Gene	Log_2_ Fold Change	Adjusted *p* Value of SDE	HR of OS	*p* Value of OS
*RAD51*	1.02	5.92 × 10^−11^	1.88	0.36
*RPA4*	1.18	1.83 × 10^−6^	2.15	0.26
*SLX1B*	1.02	2.2 × 10^−3^	1.25	0.73
*POLN*	1.00	7.16 × 10^−19^	1.99	0.30
*EME2*	1.29	1.16 × 10^−18^	5.67	0.07
*EXO1*	1.27	2.82 × 10^−11^	1.75	0.51
*DNTT*	−1.20	1.1 × 10^−2^	1.22	0.85
*NEIL3*	1.93	1.21 × 10^−18^	2.01	0.31

**Table 2:** Multivariate Cox regression analysis for OS and PFS.

**Variables for OS**	**HR**	**Lower 95% CI**	**Higher 95% CI**	***p* Value**
4-gene expression				
Lower	ref	ref	ref	ref
Higher	13.8	1.21	158	0.0348
Age (year)	1.05	0.95	1.15	0.3667
PSA (ng/mL)	0.99	0.99	1.00	0.2950
TNM stages				
T1N0M0 or T2N0M0	ref	ref	ref	ref
Others	7.27	0.92	57.39	0.0597
Gleason scores				
≤7	ref	ref	ref	ref
>7	0.72	0.14	3.62	0.6897
**Variables for PFS**	**HR**	**Lower 95% CI**	**Higher 95% CI**	***p* Value**
4-gene expression				
Lower	ref	ref	ref	ref
Higher	2.32	1.39	3.95	0.0020
Age (year)	1.00	0.98	1.04	0.6517
PSA (ng/mL)	0.99	0.99	1.00	0.2380
TNM stages				
T1N0M0 or T2N0M0	ref	ref	ref	ref
Others	1.33	0.86	2.08	0.2034
Gleason scores				
≤7	ref	ref	ref	ref
>7	2.70	1.61	4.56	0.0002

ref: reference group.

## Data Availability

Data will be provided on request.

## Supplementary Materials

The following are available online at https://www.mdpi.com/article/10.3390/ijms222111771/s1. Figure S1: Functional annotation of the 8 SDE genes based on Reactome, Figure S2: Kaplan–Meier plot of OS for individual genes, Table S1: Six DNA-repair relevant pathways and genes, Table S2: Patient characteristics.
