# Supplementary material for: Identification of DNA Damage Repair-Associated Prognostic Biomarkers for Prostate Cancer Using Transcriptomic Data Analysis
Source: Int J Mol Sci. 2021 Oct 29;22(21):11771. doi: 10.3390/ijms222111771 (PMC8584064; doi:10.3390/ijms222111771)
Supplement: Supplementary file 1 [file ijms-22-11771-s001.zip › ijms-1414547-supplementary.pdf]

Supplementary materials

Pai-Chi Teng [1], Shu-Pin Huang [2,3,4,5], Chia-Hsin Liu [6], Ting-Yi Lin [7], Yi-Chun Cho [6], Yo-Liang Lai [8,9], Shu-Chi Wang [10], Hsin-Chih Yeh [2,11], Chih-Pin Chuu [12], Deng-Neng Chen [12], Wei-Chung Cheng [6,8,13,*] and Chia-Yang Li [14,*]

[1] Taipei City Hospital Renai Branch, Taipei 10629, Taiwan; paichi.teng@gmail.com
[2] Department of Urology, School of Medicine, College of Medicine, Kaohsiung Medical University, Kaohsiung 80708, Taiwan; shpihu73@gmail.com (S.-P.H.); patrick1201.tw@yahoo.com.tw (H.-C.Y.)
[3] Department of Urology, Kaohsiung Medical University Hospital, Kaohsiung Medical University, Kaohsiung 80708, Taiwan
[4] Graduate Institute of Clinical Medicine, College of Medicine, Kaohsiung Medical University, Kaohsiung 80708, Taiwan
[5] Ph.D. Program in Environmental and Occupational Medicine, College of Medicine, Kaohsiung Medical
University, Kaohsiung 80708, Taiwan
[6] Research Center for Cancer Biology, China Medical University, Taichung 40403, Taiwan; b881642@gmail.com (C.-H.L.); demicho.0111@gmail.com (Y.-C.C.)
[7] Department of Medical Research, Taipei Veterans General Hospital, Taipei 11217, Taiwan; lintingyi2014@gmail.com
[8] Graduate Institute of Biomedical Science, China Medical University, Taichung 40403, Taiwan;
yolianglai@gmail.com
[9] Department of Radiation Oncology, China Medical University Hospital, Taichung 40403, Taiwan
[10] Department of Medical Laboratory Science and Biotechnology, Kaohsiung Medical University,
Kaohsiung 80708, Taiwan; shuchiwang@kmu.edu.tw
[11] Department of Urology, Kaohsiung Municipal Ta-Tung Hospital, Kaohsiung 80145, Taiwan; patrick1201.tw@yahoo.com.tw

[11] Institute of Cellular and System Medicine, National Health Research Institutes, Miaoli 350401, Taiwan; cpchuu@nhri.edu.tw
[12] Department Management Information Systems, National Pingtung University of Science and Technology, Pingtung 912301, Taiwan; dnchen@mail.npust.edu.tw
[13] Ph.D. Program for Cancer Biology and Drug Discovery, China Medical University and Academia Sinica, Taichung 40403, Taiwan
[14] Graduate Institute of Medicine, College of Medicine, Kaohsiung Medical University, Kaohsiung 80708, Taiwan
* Correspondence: shpihu73@gmail.com (S.-P.H.); wccheng@mail.cmu.edu.tw (W.-C.C.); chiayangli@kmu.edu.tw (C.-Y.L.)

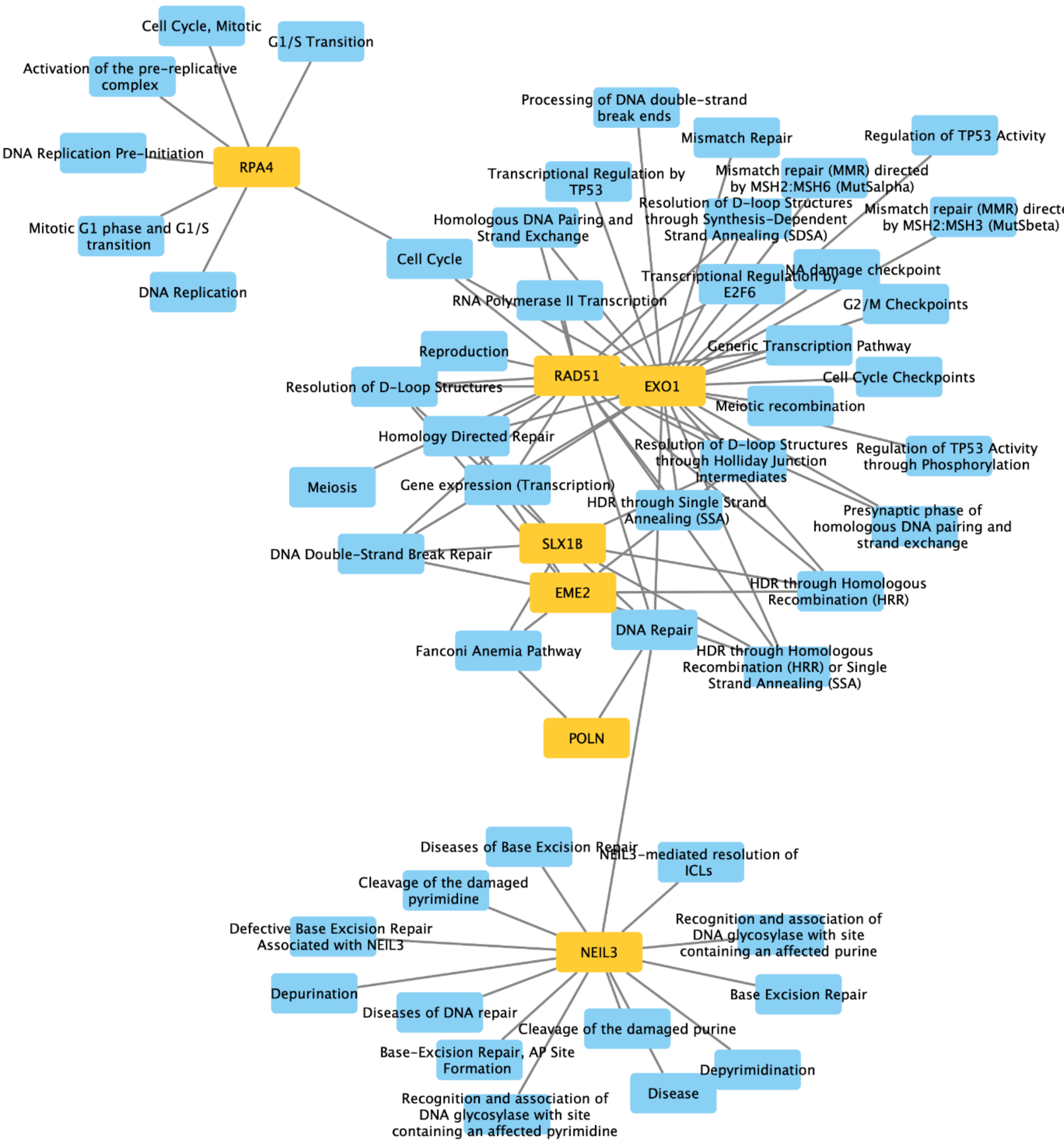

Supplementary Figure S1. Functional annotation of the 8 SDE genes based on Reactome.

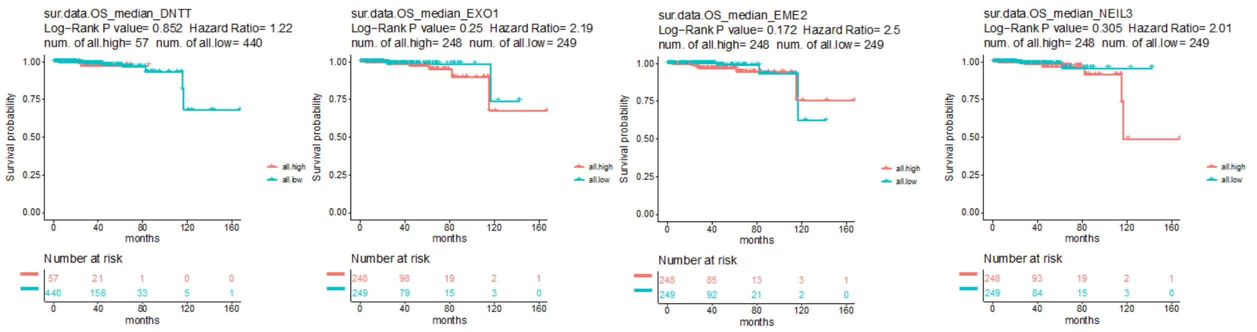

Supplementary Figure S2. Kaplan-Meier plot of OS for individual genes.

Supplementary Table S1. Six DNA-repair relevant pathways and genes.

KEGG pathway entry	KEGG pathway name	Link
hsa03410	Base excision repair - Homo sapiens (human)	https://www.genome.jp/dbget-bin/www_bget?pathway:hsa03410
hsa03420	Nucleotide excision repair - Homo sapiens (human)	https://www.genome.jp/dbget-bin/www_bget?pathway:hsa03420
hsa03430	Mismatch repair - Homo sapiens (human)	https://www.genome.jp/dbget-bin/www_bget?pathway:hsa03430
hsa03440	Homologous recombination - Homo sapiens (human)	https://www.genome.jp/dbget-bin/www_bget?pathway:hsa03440
hsa03450	Non-homologous end-joining - Homo sapiens (human)	https://www.genome.jp/dbget-bin/www_bget?pathway:hsa03450
hsa03460	Fanconi anemia pathway - Homo sapiens (human)	https://www.genome.jp/dbget-bin/www_bget?pathway:hsa03460

KEGG Gene entry	Gene Symbol	KEGG Gene Description
10038	PARP2	poly(ADP-ribose) polymerase 2 [KO:K10798] [EC:2.4.2.30]
10039	PARP3	poly(ADP-ribose) polymerase family member 3 [KO:K10798] [EC:2.4.2.30]
100526739	CENPS-CORT	CENPS-CORT readthrough [KO:K11511]
100533467	BIVM-ERCC5	BIVM-ERCC5 readthrough [KO:K10846]
10111	RAD50	RAD50 double strand break repair protein [KO:K10866] [EC:3.6.-.-]
1022	CDK7	cyclin dependent kinase 7 [KO:K02202] [EC:2.7.11.22 2.7.11.23]
1069	CETN2	centrin 2 [KO:K10840]
10714	POLD3	DNA polymerase delta 3, accessory subunit [KO:K03504]

11073	TOPBP1	DNA topoisomerase II binding protein 1 [KO:K10728]
11201	POLI	DNA polymerase iota [KO:K03510] [EC:2.7.7.7]
116028	RMI2	RecQ mediated genome instability 2 [KO:K15365]
1161	ERCC8	ERCC excision repair 8, CSA ubiquitin ligase complex subunit [KO:K10570]
142	PARP1	poly(ADP-ribose) polymerase 1 [KO:K24070] [EC:2.4.2.30]
143	PARP4	poly(ADP-ribose) polymerase family member 4 [KO:K10798] [EC:2.4.2.30]
146956	EME1	essential meiotic structure-specific endonuclease 1 [KO:K10882] [EC:3.1.22.-]
1642	DDB1	damage specific DNA binding protein 1 [KO:K10610]
1643	DDB2	damage specific DNA binding protein 2 [KO:K10140]
1791	DNTT	DNA nucleotidylexotransferase [KO:K00977] [EC:2.7.7.31]
197342	EME2	essential meiotic structure-specific endonuclease subunit 2 [KO:K10883]
201254	CENPX	centromere protein X [KO:K15360]
2067	ERCC1	ERCC excision repair 1, endonuclease non-catalytic subunit [KO:K10849]
2068	ERCC2	ERCC excision repair 2, TFIIH core complex helicase subunit [KO:K10844] [EC:3.6.4.12]
2071	ERCC3	ERCC excision repair 3, TFIIH core complex helicase subunit [KO:K10843] [EC:3.6.4.12]
2072	ERCC4	ERCC excision repair 4, endonuclease catalytic subunit [KO:K10848] [EC:3.1.-.-]
2073	ERCC5	ERCC excision repair 5, endonuclease [KO:K10846]
2074	ERCC6	ERCC excision repair 6, chromatin remodeling factor [KO:K10841]
2175	FANCA	FA complementation group A [KO:K10888]
2176	FANCC	FA complementation group C [KO:K10890]
2177	FANCD2	FA complementation group D2 [KO:K10891]
2178	FANCE	FA complementation group E [KO:K10892]
2187	FANCB	FA complementation group B [KO:K10889]
2188	FANCF	FA complementation group F [KO:K10893]
2189	FANCG	FA complementation group G [KO:K10894]
2237	FEN1	flap structure-specific endonuclease 1 [KO:K04799] [EC:3.-.-.-]
22909	FAN1	FANCD2 and FANCI associated nuclease 1 [KO:K15363] [EC:3.1.21.- 3.1.4.1]
23583	SMUG1	single-strand-selective monofunctional uracil-DNA glycosylase 1 [KO:K10800] [EC:3.2.2.-]

252969	NEIL2	nei like DNA glycosylase 2 [KO:K10568] [EC:3.2.2.- 4.2.99.18]
2547	XRCC6	X-ray repair cross complementing 6 [KO:K10884]
25788	RAD54B	RAD54 homolog B [KO:K10877] [EC:3.6.4.-]
27030	MLH3	mutL homolog 3 [KO:K08739]
27301	APEX2	apurinic/apyrimidinic endodeoxyribonuclease 2 [KO:K10772] [EC:4.2.99.18]
27343	POLL	DNA polymerase lambda [KO:K03512] [EC:2.7.7.7 4.2.99.-]
27434	POLM	DNA polymerase mu [KO:K03513] [EC:2.7.7.7]
29086	BABAM1	BRISC and BRCA1 A complex member 1 [KO:K20776]
29089	UBE2T	ubiquitin conjugating enzyme E2 T [KO:K13960] [EC:2.3.2.23]
2956	MSH6	mutS homolog 6 [KO:K08737]
2965	GTF2H1	general transcription factor IIH subunit 1 [KO:K03141]
2966	GTF2H2	general transcription factor IIH subunit 2 [KO:K03142]
2967	GTF2H3	general transcription factor IIH subunit 3 [KO:K03143]
2968	GTF2H4	general transcription factor IIH subunit 4 [KO:K03144]
29935	RPA4	replication protein A4 [KO:K10741]
3146	HMGB1	high mobility group box 1 [KO:K10802]
328	APEX1	apurinic/apyrimidinic endodeoxyribonuclease 1 [KO:K10771] [EC:4.2.99.18]
3280	HES1	hes family bHLH transcription factor 1 [KO:K06054]
353497	POLN	DNA polymerase nu [KO:K16618] [EC:2.7.7.7]
378708	CENPS	centromere protein S [KO:K11511]
3978	LIG1	DNA ligase 1 [KO:K10747] [EC:6.5.1.1 6.5.1.6 6.5.1.7]
3980	LIG3	DNA ligase 3 [KO:K10776] [EC:6.5.1.1]
3981	LIG4	DNA ligase 4 [KO:K10777] [EC:6.5.1.1]
404672	GTF2H5	general transcription factor IIH subunit 5 [KO:K10845]
4292	MLH1	mutL homolog 1 [KO:K08734]
4331	MNAT1	MNAT1 component of CDK activating kinase [KO:K10842]
4350	MPG	N-methylpurine DNA glycosylase [KO:K03652] [EC:3.2.2.21]
4361	MRE11	MRE11 homolog, double strand break repair nuclease [KO:K10865]
4436	MSH2	mutS homolog 2 [KO:K08735]
4437	MSH3	mutS homolog 3 [KO:K08736]

4595	MUTYH	mutY DNA glycosylase [KO:K03575] [EC:3.2.2.31]
4683	NBN	nibrin [KO:K10867]
472	ATM	ATM serine/threonine kinase [KO:K04728] [EC:2.7.11.1]
4913	NTHL1	nth like DNA glycosylase 1 [KO:K10773] [EC:4.2.99.18]
4968	OGG1	8-oxoguanine DNA glycosylase [KO:K03660] [EC:3.2.2.- 4.2.99.18]
50511	SYCP3	synaptonemal complex protein 3 [KO:K19528]
5111	PCNA	proliferating cell nuclear antigen [KO:K04802]
51426	POLK	DNA polymerase kappa [KO:K03511] [EC:2.7.7.7]
51455	REV1	REV1 DNA directed polymerase [KO:K03515] [EC:2.7.7.-]
51720	UIMC1	ubiquitin interaction motif containing 1 [KO:K20775]
5395	PMS2	PMS1 homolog 2, mismatch repair system component [KO:K10858]
54107	POLE3	DNA polymerase epsilon 3, accessory subunit [KO:K02326] [EC:2.7.7.7]
5423	POLB	DNA polymerase beta [KO:K02330] [EC:2.7.7.7 4.2.99.-]
5424	POLD1	DNA polymerase delta 1, catalytic subunit [KO:K02327] [EC:2.7.7.7]
5425	POLD2	DNA polymerase delta 2, accessory subunit [KO:K02328]
5426	POLE	DNA polymerase epsilon, catalytic subunit [KO:K02324] [EC:2.7.7.7]
5427	POLE2	DNA polymerase epsilon 2, accessory subunit [KO:K02325] [EC:2.7.7.7]
5429	POLH	DNA polymerase eta [KO:K03509] [EC:2.7.7.7]
545	ATR	ATR serine/threonine kinase [KO:K06640] [EC:2.7.11.1]
548593	SLX1A	SLX1 homolog A, structure-specific endonuclease subunit [KO:K15078] [EC:3.6.1.-]
55120	FANCL	FA complementation group L [KO:K10606] [EC:2.3.2.27]
55215	FANCI	FA complementation group I [KO:K10895]
55247	NEIL3	nei like DNA glycosylase 3 [KO:K10569]
5591	PRKDC	protein kinase, DNA-activated, catalytic subunit [KO:K06642] [EC:2.7.11.1]
56655	POLE4	DNA polymerase epsilon 4, accessory subunit [KO:K03506] [EC:2.7.7.7]
57599	WDR48	WD repeat domain 48 [KO:K15361]
57697	FANCM	FA complementation group M [KO:K10896]
57804	POLD4	DNA polymerase delta 4, accessory subunit [KO:K03505]
580	BARD1	BRCA1 associated RING domain 1 [KO:K10683]

5886	RAD23A	RAD23 homolog A, nucleotide excision repair protein [KO:K10839]
5887	RAD23B	RAD23 homolog B, nucleotide excision repair protein [KO:K10839]
5888	RAD51	RAD51 recombinase [KO:K04482]
5889	RAD51C	RAD51 paralog C [KO:K10870]
5890	RAD51B	RAD51 paralog B [KO:K10869]
5892	RAD51D	RAD51 paralog D [KO:K10871]
5893	RAD52	RAD52 homolog, DNA repair protein [KO:K10873]
5932	RBBP8	RB binding protein 8, endonuclease [KO:K20773] [EC:3.1.-.-]
5980	REV3L	REV3 like, DNA directed polymerase zeta catalytic subunit [KO:K02350] [EC:2.7.7.7]
5981	RFC1	replication factor C subunit 1 [KO:K10754]
5982	RFC2	replication factor C subunit 2 [KO:K10755]
5983	RFC3	replication factor C subunit 3 [KO:K10756]
5984	RFC4	replication factor C subunit 4 [KO:K10755]
5985	RFC5	replication factor C subunit 5 [KO:K10756]
6117	RPA1	replication protein A1 [KO:K07466]
6118	RPA2	replication protein A2 [KO:K10739]
6119	RPA3	replication protein A3 [KO:K10740]
641	BLM	BLM RecQ like helicase [KO:K10901] [EC:3.6.4.12]
64421	DCLRE1C	DNA cross-link repair 1C [KO:K10887] [EC:3.1.-.-]
672	BRCA1	BRCA1 DNA repair associated [KO:K10605] [EC:2.3.2.27]
6742	SSBP1	single stranded DNA binding protein 1 [KO:K03111]
675	BRCA2	BRCA2 DNA repair associated [KO:K08775]
6996	TDG	thymine DNA glycosylase [KO:K20813] [EC:3.2.2.29]
7156	TOP3A	DNA topoisomerase III alpha [KO:K03165] [EC:5.6.2.1]
728340	GTF2H2C	GTF2H2 family member C [KO:K03142]
730394	GTF2H2C_2	GTF2H2 family member C, copy 2 [KO:K03142]
7374	UNG	uracil DNA glycosylase [KO:K03648] [EC:3.2.2.27]
7398	USP1	ubiquitin specific peptidase 1 [KO:K11832] [EC:3.4.19.12]
7507	XPA	XPA, DNA damage recognition and repair factor [KO:K10847]
7508	XPC	XPC complex subunit, DNA damage recognition and repair factor [KO:K10838]
7515	XRCC1	X-ray repair cross complementing 1 [KO:K10803]
7516	XRCC2	X-ray repair cross complementing 2 [KO:K10879]
7517	XRCC3	X-ray repair cross complementing 3 [KO:K10880]

7518	XRCC4	X-ray repair cross complementing 4 [KO:K10886]
7520	XRCC5	X-ray repair cross complementing 5 [KO:K10885]
79008	SLX1B	SLX1 homolog B, structure-specific endonuclease subunit [KO:K15078] [EC:3.6.1.-]
79184	BRCC3	BRCA1/BRCA2-containing complex subunit 3 [KO:K11864] [EC:3.4.19.-]
79661	NEIL1	nei like DNA glycosylase 1 [KO:K10567] [EC:3.2.2.- 4.2.99.18]
79728	PALB2	partner and localizer of BRCA2 [KO:K10897]
7979	SEM1	SEM1 26S proteasome subunit [KO:K10881]
79840	NHEJ1	non-homologous end joining factor 1 [KO:K10980]
80010	RMI1	RecQ mediated genome instability 1 [KO:K10990]
80198	MUS81	MUS81 structure-specific endonuclease subunit [KO:K08991] [EC:3.1.22.-]
80233	FAAP100	FA core complex associated protein 100 [KO:K10993]
83990	BRIP1	BRCA1 interacting helicase 1 [KO:K15362] [EC:3.6.4.12]
84126	ATRIP	ATR interacting protein [KO:K10905]
84142	ABRAXAS1	abraxas 1, BRCA1 A complex subunit [KO:K20774]
8438	RAD54L	RAD54 like [KO:K10875] [EC:3.6.4.-]
84464	SLX4	SLX4 structure-specific endonuclease subunit [KO:K10484]
8450	CUL4B	cullin 4B [KO:K10609]
8451	CUL4A	cullin 4A [KO:K10609]
8930	MBD4	methyl-CpG binding domain 4, DNA glycosylase [KO:K10801] [EC:3.2.2.-]
8940	TOP3B	DNA topoisomerase III beta [KO:K03165] [EC:5.6.2.1]
902	CCNH	cyclin H [KO:K06634]
91442	FAAP24	FA core complex associated protein 24 [KO:K10898]
9156	EXO1	exonuclease 1 [KO:K10746] [EC:3.1.-.-]
9577	BABAM2	BRISC and BRCA1 A complex member 2 [KO:K12173]
9894	TELO2	telomere maintenance 2 [KO:K11137]
9978	RBX1	ring-box 1 [KO:K03868] [EC:2.3.2.32]

Supplementary Table S2. Patient characteristics.

**Number of patients**	497
**Age (year)**	
Mean	61.61
standard deviation	6.79
**Gleason scores**	
6	45
7	247
8	64
9	137
10	4
**TNM stage**	
T1N0M0 or T2N0M0	252
others	245
